# Effects of laughter therapy on quality of life in patients with cancer: An open-label, randomized controlled trial

**DOI:** 10.1371/journal.pone.0219065

**Published:** 2019-06-27

**Authors:** Toshitaka Morishima, Isao Miyashiro, Norimitsu Inoue, Mitsuko Kitasaka, Takashi Akazawa, Akemi Higeno, Atsushi Idota, Akira Sato, Tetsuya Ohira, Masato Sakon, Nariaki Matsuura

**Affiliations:** 1 Cancer Control Center, Osaka International Cancer Institute, Osaka, Japan; 2 Department of Tumor Immunology, Research Center, Osaka International Cancer Institute, Osaka, Japan; 3 Department of Molecular Genetics, Wakayama Medical University, Wakayama, Japan; 4 Department of Nursing Services, Osaka International Cancer Institute, Osaka, Japan; 5 Department of Cancer Drug Discovery and Development, Research Center, Osaka International Cancer Institute, Osaka, Japan; 6 Department of Clinical Laboratory, Osaka International Cancer Institute, Osaka, Japan; 7 Department of Epidemiology, Fukushima Medical University, Fukushima, Japan; 8 Osaka International Cancer Institute, Osaka, Japan; Vanderbilt University, UNITED STATES

## Abstract

**Background:**

Few randomized controlled trials have assessed the effects of laughter therapy on health-related quality of life (QOL) in cancer patients. This study aimed to evaluate these effects as an exploratory endpoint in cancer patients as part of a randomized controlled trial conducted at a single institution in Japan.

**Methods:**

The Initiative On Smile And CAncer (iOSACA) study was an open-label randomized controlled trial conducted in 2017 in which participants aged 40–64 years with cancer were randomly assigned to either an intervention group (laughter therapy) or control group (no laughter therapy). Each participant in the intervention group underwent a laughter therapy session once every two weeks for seven weeks (total of four sessions). Each session involved a laughter yoga routine followed by *Rakugo* or *Manzai* traditional Japanese verbal comedy performances. We assessed QOL as a secondary endpoint in this intention-to-treat population using the European Organisation for Research and Treatment of Cancer Quality of Life Questionnaire Core 30 (EORTC QLQ-C30). The questionnaire was completed at baseline (Week 0) and at Weeks 3 and 7. Mixed-effects models for repeated measures were developed to compare time-dependent changes in each QOL domain from baseline between the intervention and control groups.

**Results:**

Four participants retracted consent and one participant was retrospectively excluded from analysis due to unmet inclusion criteria. The analysis was conducted using 56 participants, with 26 in the intervention group and 30 in the control group. Questionnaire completion rates were high (>90%), with similar QOL scores reported at baseline in both groups. The mixed-effects models showed that the intervention group had significantly better cognitive function and less pain than the control group for a short period.

**Conclusion:**

Laughter therapy may represent a beneficial, noninvasive complementary intervention in the clinical setting. Further studies are needed to verify the hypotheses generated from this exploratory study.

## Introduction

Over the past 20 years, widespread screening and advances in treatment options have allowed individuals to live longer after cancer diagnosis [1]. This has led to a continuously growing population of cancer survivors living with various sequelae [2]. The impact of cancer on health-related quality of life (QOL) during the acute diagnostic and treatment phases is well documented [3]. Moreover, attention has also been directed toward cancer’s longer-term physical and psycho-social effects. Cancer survivors are more likely to suffer from secondary health problems such as impaired cognitive function, fatigue, pain, anxiety, and depression [4–6]. This may have an impact on their QOL, which is generally lower than that of the general population [7].

Effective interventions are therefore needed to reduce QOL impairment in cancer patients. In addition to conventional treatments, complementary and alternative therapies have been developed to improve QOL and manage symptoms [8]. Laughter therapy (which often includes laughter yoga, comedy performances, clown performances, and jokes) has been applied as a complementary intervention since the 1970s [9]. Laughter therapy can benefit health through various mechanisms, including muscular exercise, increased respiration and blood circulation, improvements to digestion, and emotional catharsis [10]. Researchers have since investigated the therapeutic efficacy of this therapy, and reported that it can have positive and quantifiable effects on a variety of medical conditions, such as depression, anxiety, stress, dementia, and pain without deleterious effects on health [11–24]. Furthermore, researchers have also reported that laughter therapy can reduce the levels of depression, anxiety, and stress in cancer patients [25–29]. However, the causal effects of laughter therapy in cancer patients remain unclear because the majority of previous studies were conducted without comparisons or randomized treatment allocation. Several randomized controlled clinical trials have been conducted to examine the therapeutic efficacy of laughter therapy in patients with breast cancer, gastrointestinal cancer, and leukemia [30–34], but these have generally focused on psychological outcomes rather than physiological ones.

The goal of active cancer therapy is to improve survival and QOL in patients [35]. QOL reflects the impact of long-term physical and psychological sequelae in cancer survivors. The American Cancer Society, which defines cancer survivorship as beginning at cancer diagnosis and continuing for the balance of life, views QOL as a key outcome of survivorship [36]. When examining the effects of integrative, alternative, and complementary therapies on cancer patients, QOL is recommended in the US National Cancer Institute’s Physician Data Query cancer information summaries as a scientifically strong endpoint for clinical trials [37]. Nevertheless, few randomized controlled studies have investigated the effects of laughter therapy on QOL [34]. To address this issue, we examined, as part of a randomized controlled trial, whether laughter therapy would improve QOL in cancer survivors. As this study was exploratory, the analysis was conducted without a specific a priori hypothesis.

## Methods

### Study design and participants

The Initiative On Smile And CAncer (iOSACA) study was a single-center, open-label, cross-over, randomized controlled trial designed to evaluate the effects of laughter therapy in cancer patients. The iOSACA study also included a prospective single-arm trial to evaluate the feasibility of four-month-long laughter therapy in cancer patients, and a randomized clinical trial to evaluate the psychological effects on work stress in hospital staff; these aspects of the study will be reported elsewhere. Patients receiving active treatment or follow-up for cancer were recruited from outpatient clinics at Osaka International Cancer Institute (OICI), Osaka, Japan in April 2017. The eligibility criteria for participants included having histologic and/or cytologic evidence of any malignancy; being aged 40 to 64 years; having an Eastern Cooperative Oncology Group (ECOG) performance status (PS) of 0 (indicating a fully active patient), 1 (indicating an ambulatory patient with restrictions on strenuous activities), or 2 (indicating an ambulatory patient capable of self-care but unable to work); and having an anticipated survival time of at least one year. Patients were excluded if they did not understand Japanese or had plans to undergo cancer surgery.

Prospective participants were informed of the risks and benefits of study participation, and provided written informed consent before enrollment. Participants were randomly assigned to two groups (Group A and Group B) in a 1:1 ratio according to a computer-generated sequence. Randomization with minimization was performed with stratifications for sex, cancer site (breast, gastrointestinal, lung, urological or gynecological, and others), and whether a patient was receiving chemotherapy or radiotherapy at the time of enrollment [38]. This study was approved by the institutional review board of OICI (Approval number: 1702246289) and registered in the UMIN Clinical Trial Registry (UMIN000026831) on April 3, 2017 (https://upload.umin.ac.jp/cgi-open-bin/ctr/ctr_view.cgi?recptno=R000030790). The CONSORT checklist and protocol for this trial are available as supporting information (S1–S3 Files).

### Intervention

Table 1 outlines the schedule of the intervention and assessments. This study consisted of two phases: a seven-week initial phase (including the baseline measurement) and a six-week cross-over phase. During the initial phase, Group A was designated the intervention group and the participants underwent laughter therapy in Weeks 1, 3, 5, and 7. Participants in Group B, which served as the control group during this phase, received no laughter therapy. The initial phase was followed by the cross-over phase from Weeks 9 through 15, during which participants in Group B crossed over to receive laughter therapy in Weeks 9, 11, 13, and 15. Group A served as the control group during the cross-over phase. The initial phase started in May 2017, and the cross-over phase ended in August 2017. The primary interest of this analysis was the initial phase, in which we investigated the effects of laughter therapy that Group A (intervention) participants received when compared with Group B (control) participants. The cross-over phase was designed to enable further analyses to determine if the effects of laughter therapy were maintained after treatment cessation and to detect any differences between carry-over effects in Group A and current effects in Group B.

**Table 1:** Schedule of laughter therapy and assessments.

	Study week									
	-6 to -2	0	1	3	5	7	9	11	13	15
	Enrollment	Baseline	Initial phase				Cross-over phase			
Laughter therapy										
Group A			x	x	x	x				
Group B							x	x	x	x
Assessments^a^										
Quality of life		x		x		x		x		x
ECOG PS		x								
Ongoing cancer treatment	x									

Abbreviations: ECOG, Eastern Cooperative Oncology Group; PS, performance status.

^a^Assessments were performed for both groups according to the same schedule.

Participants assigned to the intervention group underwent a one-hour laughter therapy session every two weeks over the course of seven weeks, with each participant receiving a total of four sessions. Each session was performed at OICI, and began with a laughter yoga routine (a group practice involving voluntary laughter, with a body exercise which includes stretching, clapping, and body movement). This was followed by live performances of *Rakugo* (a form of Japanese verbal comedy performed by a lone storyteller sitting on stage) or *Manzai* (a traditional Japanese style of stand-up comedy involving jokes traded at high speed between two performers) by locally well-known professional entertainers.

### QOL assessments

This study was conducted to investigate the effects of laughter therapy on QOL as a secondary endpoint of the iOSACA study. The primary endpoint was self-efficacy, which is not addressed in this paper. QOL was measured using the European Organisation for Research and Treatment of Cancer Quality of Life Questionnaire Core 30 version 3.0 (EORTC QLQ-C30), a validated questionnaire designed to assess both disease-related functions and symptoms in addition to their impact on everyday life [39]. The time frame for each assessment was the immediately preceding week, which may be of particular relevance to clinical trials.

The QLQ-C30 is a 30-item, cancer-specific, multi-dimensional, self-administered questionnaire that has been validated in cross-cultural settings [40]. It comprises a two-item global health status domain; five multi-item functional domains (physical functioning, role functioning, emotional functioning, cognitive functioning, and social functioning); three multi-item symptom domains (fatigue, pain, and nausea and vomiting); and six single-item domains for the assessment of additional symptoms commonly reported by cancer patients (dyspnea, appetite loss, sleep disturbance, constipation, and diarrhea) and the perceived financial impact of the disease and treatment. Most items are answered based on a four-point Likert scale ranging from 1 (not at all) to 4 (very much). Responses to the two items assessing global health status range from 1 (very poor) to 7 (excellent). Items were scaled and scored according to the EORTC Scoring Manual [41]. Raw scores were transformed to a linear scale ranging from 0 to 100 [41]. For scores measuring global health status and functional domains, a higher score represents a ‘better’ level of status or functioning; in contrast, a higher score for symptom domains represents a ‘worse’ level of symptoms.

Questionnaires were completed on Week 0 (baseline, one week before the start of the intervention) and every four weeks during both the initial and cross-over phases. Participants undergoing the intervention self-administered the questionnaire immediately after a laughter therapy session at OICI, whereas control participants self-administered the questionnaire when visiting OICI during the same scheduled time points.

### Statistical analyses

We calculated that 30 participants would be required for each group based on a sample size estimation for the iOSACA study’s primary endpoint (i.e., self-efficacy). Questionnaire completion rates for each group were calculated as the percentage of all subjects who completed a questionnaire at each scheduled time point. Primary analyses were performed using three assessments of the QOL domains obtained at baseline and during the initial phase. Statistical analyses of all 15 QOL domain scores were used to evaluate the differences between the groups with respect to changes from the baseline scores to those obtained at each assessment point as follows: changes from baseline to Week 3 in Group A versus Group B and changes from baseline to Week 7 in Group A versus Group B. Between-group differences in these time-dependent changes from baseline and their 95% confidence intervals (CIs) were analyzed using mixed-effects models for repeated measures in which the covariates were laughter therapy during each phase (receiving laughter therapy or not), time point, therapy phase (receiving laughter therapy in the initial or cross-over phase), therapy-by-time point interactions, and the three predefined stratification factors (sex, cancer site, and receiving chemotherapy or radiotherapy at the time of enrollment) as fixed effects [42–45]. Unstructured covariance matrices were used for these analyses. No correlation structures of residual error terms were specified.

A secondary analysis of within-group differences was performed for each QOL domain to examine if the effects of laughter therapy were maintained after the intervention was stopped. For these analyses, changes from the end of the initial phase (i.e., the score of Week 7) to the cross-over phase (i.e., the score of Week 11 and the score of Week 15) in Group A were examined. Another secondary analysis was conducted to detect between-group differences in each QOL domain for the carry-over effects and current effects of laughter therapy. For these analyses, the between-group comparisons of changes from baseline to each assessment point during the cross-over phase were conducted as follows: changes from baseline to Week 11 in Group A versus Group B and changes from baseline to Week 15 in Group A versus Group B. All comparisons between the groups were based on intention-to-treat analyses in which participants were analyzed according to their assigned group. These analyses were not pre-specified in the trial protocol. All statistical analyses were performed using SAS version 9.4 (SAS Institute, Cary, NC, USA). All statistical tests were two-sided, and *P* values below 0.05 were considered statistically significant.

## Results

### Participant characteristics

**Fig 1:**
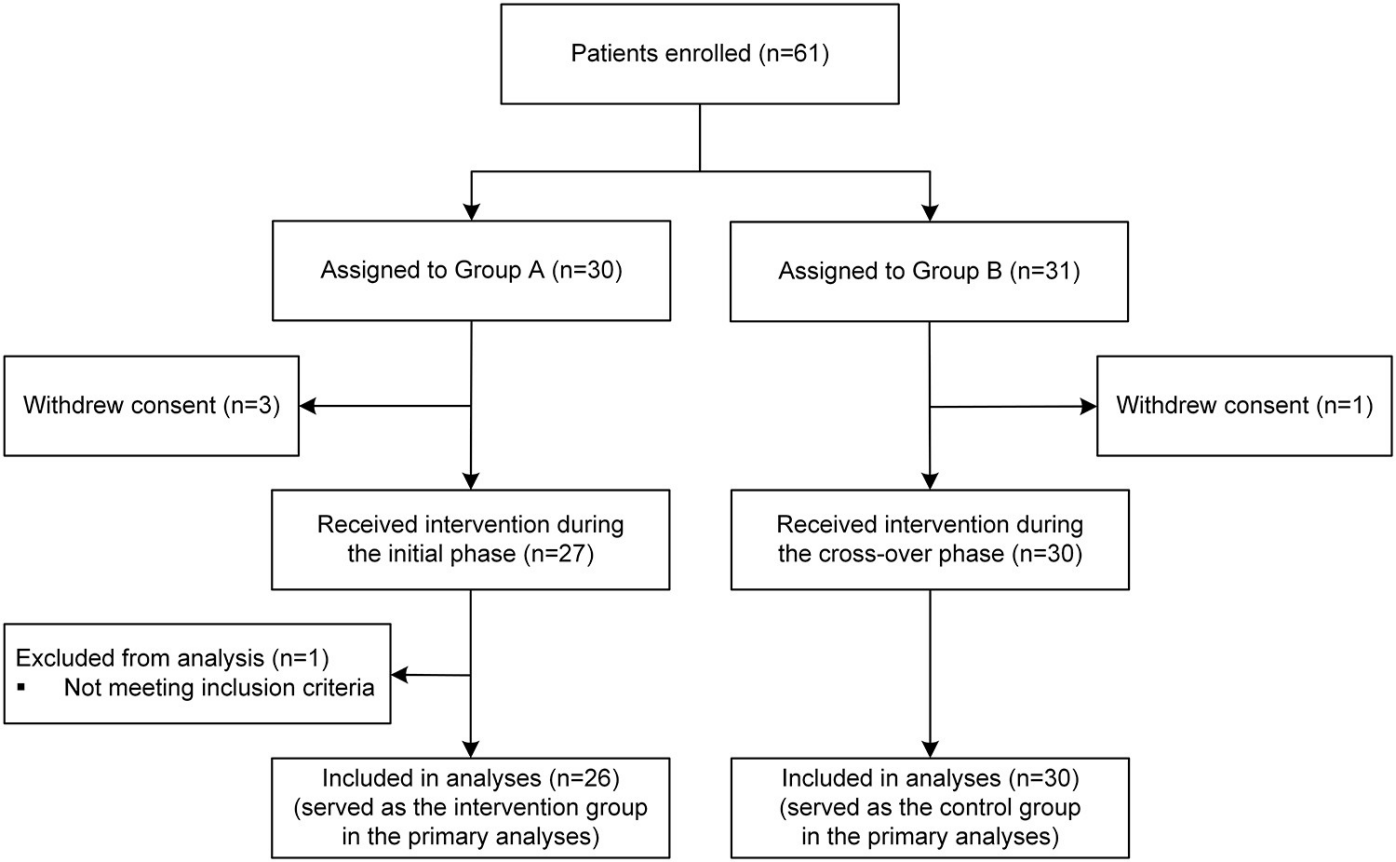
Study participant flow diagram.

A flow diagram of this clinical trial is presented in Fig 1. Sixty-one patients provided consent to be enrolled in the trial. Thirty participants were randomized to Group A and the remaining 31 participants formed Group B. However, four participants withdrew their consent. One participant was retrospectively excluded from the analyses because (s)he was found not to have met the age inclusion criterion through data curation after the trial. In total, 56 participants were enrolled (26 allocated to Group A and 30 to Group B) and included in the analyses.

The demographic and medical characteristics of the 56 participants are summarized in Table 2. These characteristics were generally well balanced between the two groups. The participants comprised a higher proportion of women (75%) than men. Breast cancer (48%) was the most common cancer site. Each group was allocated a similar proportion of participants who were receiving chemotherapy or radiotherapy at study enrollment (approximately 34%). None of the participants had an ECOG PS of 2. Group A had a lower proportion of participants with an ECOG PS of 0 than Group B (50% versus 63%, respectively).

**Table 2:** Participant characteristics.

	Group A	Group B
Total number of participants	26 (100%)	30 (100%)
Sex		
Female	20 (77%)	22 (73%)
Age, years		
Median (IQR)	55 (48–61)	56 (52–62)
Cancer site		
Breast	14 (54%)	13 (43%)
Gastrointestinal	6 (23%)	9 (30%)
Lung	2 (8%)	2 (7%)
Urological or gynecological	1 (4%)	0 (0%)
Others	3 (12%)	6 (20%)
Chemotherapy or radiotherapy at study enrollment		
Yes	9 (35%)	10 (33%)
ECOG PS		
0	13 (50%)	19 (63%)
1	13 (50%)	11 (37%)
2	0 (0%)	0 (0%)

Abbreviations: ECOG, Eastern Cooperative Oncology Group; IQR, interquartile range; PS, performance status.

Values are expressed as the number of participants (column percentage) unless otherwise indicated.

### Laughter therapy and QOL questionnaire completion

Compliance with the laughter therapy sessions and QOL assessments is presented in Table 3. Among the participants in Group A, 25 participated in all sessions from Weeks 1 through 7, with one participant missing a session in Week 3. Among the participants in Group B, 27 participated in all sessions from Weeks 9 through 15, with one participant missing each post-baseline session.

**Table 3:** Completion of laughter therapy sessions and the EORTC QLQ-C30 assessment.

	Laughter therapy		QOL assessment	
	Group A	Group B	Group A	Group B
	(n = 26)	(n = 30)	(n = 26)	(n = 30)
Study week				
Baseline (Week 0)	—	—	26 (100%)	30 (100%)
Week 1	26 (100%)	—	—	—
Week 3	25 (96%)	—	25 (96%)	27 (90%)
Week 5	26 (100%)	—	—	—
Week 7	26 (100%)	—	26 (100%)	28 (93%)
Week 9	—	30 (100%)	—	—
Week 11	—	29 (97%)	24 (92%)	29 (97%)
Week 13	—	29 (97%)	—	—
Week 15	—	29 (97%)	26 (100%)	29 (97%)
Completed all sessions/assessments	25 (96%)	27 (90%)	23 (88%)	24 (80%)

Abbreviations: EORTC QLQ-C30, European Organisation for Research and Treatment of Cancer Quality of Life Questionnaire Core 30; QOL, quality of life.

Values are expressed as the number of participants (column percentage). A dash indicates that therapy or assessment was not performed for the column group during the designated week.

All participants in both groups completed the baseline QLQ-C30 assessment, and most participants (84%) completed all post-baseline questionnaires. Completion rates, as expressed by the percentage of eligible participants who were expected to complete the QOL questionnaires at each specific time point, ranged between 90% and 100%.

### Baseline QOL scores

The two groups had similar baseline QOL scores in all domains (Table 4). The greatest impairment was seen in global health status (mean scores of 72.1 and 77.5 in Group A and Group B, respectively), cognitive functioning (75.0 and 80.6, respectively) and emotional functioning (77.6 and 80.3, respectively). The symptoms with the highest mean baseline scores were fatigue (mean scores of 35.9 and 28.5 in Group A and Group B, respectively), insomnia (21.8 and 22.2, respectively), financial difficulties (19.2 and 15.6, respectively), and dyspnea (21.8 and 10.0, respectively).

**Table 4:** Baseline quality of life in the study participants by group.

	Group A (n = 26)	Group B (n = 30)
EORTC QLQ-C30 global health status and functional scores		
Global health status	72.1 (17.9)	77.5 (22.5)
Physical functioning	87.9 (10.2)	89.1 (9.8)
Role functioning	85.3 (17.8)	85.6 (18.4)
Emotional functioning	77.6 (16.6)	80.3 (17.4)
Cognitive functioning	75.0 (16.5)	80.6 (20.6)
Social functioning	85.3 (22.8)	83.9 (24.2)
EORTC QLQ-C30 symptom scores		
Fatigue	35.9 (18.4)	28.5 (19.3)
Nausea and vomiting	1.9 (5.4)	3.9 (9.5)
Pain	15.4 (20.5)	12.2 (19.5)
Dyspnea	21.8 (23.0)	10.0 (15.5)
Insomnia	21.8 (21.0)	22.2 (22.0)
Appetite loss	11.5 (16.2)	11.1 (18.2)
Constipation	10.3 (15.7)	13.3 (22.5)
Diarrhea	16.7 (19.4)	11.1 (22.0)
Financial difficulties	19.2 (28.6)	15.6 (25.9)

Abbreviation: EORTC QLQ-C30, European Organisation for Research and Treatment of Cancer Quality of Life Questionnaire Core 30.

Values are expressed as the mean score (standard deviation) of each group. Raw scores were transformed to a linear scale ranging from 0 to 100, with higher scores representing a higher (better) level of health status and functioning or a higher (worse) level of symptoms.

### Primary QOL analyses

**Fig 2:**
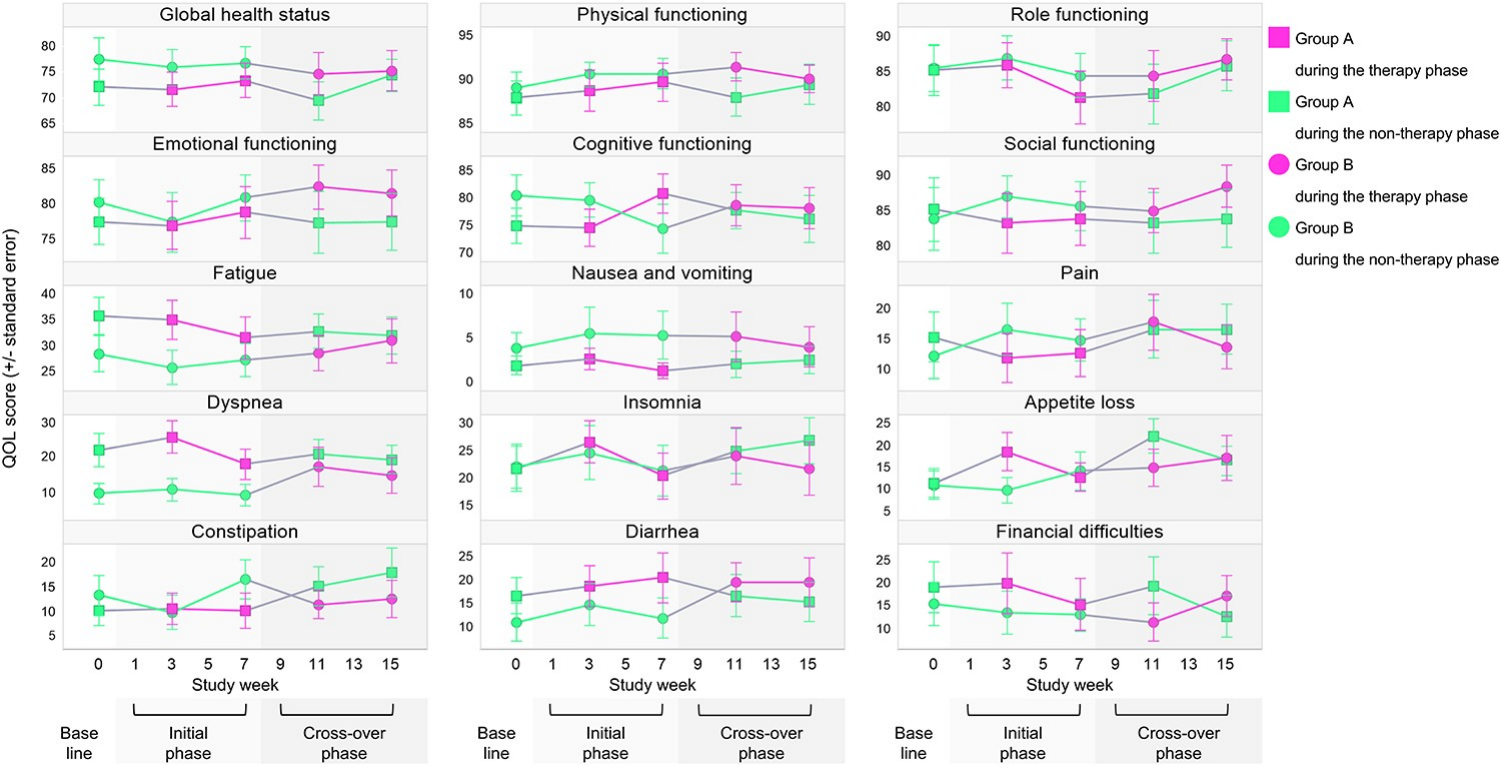
Time-dependent changes in 15 quality of life domains.

Fig 2 illustrates the time-dependent changes in the mean scores for each QLQ-C30 domain from baseline. Primary QOL analyses were performed for three assessments obtained from baseline up to Week 7 to compare these changes between the intervention group (i.e., Group A) and the control group (i.e., Group B) in the initial phase. In Group A, the global health status score had worsened by 0.3 points at Week 3 and improved by 1.3 points at Week 7 relative to the baseline score. In Group B, this score had worsened by 2.1 points at Week 3 and by 1.1 point at Week 7 relative to the baseline score. These scores did not demonstrate statistically significant between-group differences at any week (95% CI: -7.6 to 11.1 points for Week 3 and -6.9 to 11.6 points for Week 7). For functional domains, the cognitive functioning score improved by 5.8 points from baseline to Week 7 in Group A, but worsened by 5.3 points in Group B. This resulted in a significant between-group difference that favored Group A over Group B (95% CI: 3.2 to 19.0 points; *P* = 0.006). There were no significant between-group differences in the other functional domains.

For symptom domains, the pain score improved by 3.9 points from baseline to Week 3 in Group A, but worsened by 4.5 points in Group B. This resulted in a significant between-group difference that favored Group A over Group B (95% CI: -16.4 to -0.5 points; *P* = 0.037). Although a similar between-group difference of 5.1 points in the pain score was observed at Week 7, the difference did not reach statistical significance (95% CI: -12.9 to 2.7 points; *P* = 0.20). The nausea and vomiting score was generally stable over time in Group A, but worsened in Group B. Although this finding appeared to favor Group A, the observed between-group differences were not statistically significant (95% CI: -6.4 to 4.9 points for Week 3 and -7.8 to 3.4 points for Week 7). Neither group showed improvements or statistically significant between-group differences for the other symptom domains.

### Secondary QOL analyses

Fig 2 also illustrates the mean score changes in the QOL domains from the initial phase to the cross-over phase, which indicate the carry-over effects after the cessation of laughter therapy in Group A. For the global health status and functional domains, the cognitive functioning score did not change significantly from Week 7 to Week 11 (-3.7 points; 95% CI: -10.6 to 3.2 points; *P* = 0.29) or from Week 7 to Week 15 (1.0 point; 95% CI: -5.8 to 7.7 points; *P* = 0.78). Also, pain score did not change significantly from Week 7 to Week 11 (4.2 points; 95% CI: -1.7 to 10.0 points; *P* = 0.160) or from Week 7 to Week 15 (3.8 points; 95% CI: -1.8 to 9.5 points; *P* = 0.183). However, one of the symptom domains showed deterioration in Group A after the cessation of laughter therapy. The appetite loss score increased significantly from Week 7 to Week 11 by 8.2 points (95% CI: 0.5 to 15.9 points; *P* = 0.037). There were no significant changes in mean scores for the other domains from the initial phase to the cross-over phase.

Participants who did not receive laughter therapy in the initial phase (i.e., Group B) were given the opportunity to do so in the cross-over phase, whereas participants who received laughter therapy in the initial phase (i.e., Group A) ceased to receive this therapy in the cross-over phase. This design enabled us to compare the carry-over effects and current effects of laughter therapy. The results showed no significant between-group differences in the changes in any QOL domain score from baseline to Weeks 11 or 15.

Across the two phases, there were no significant between-group differences that favored the current control group over the current intervention group.

## Discussion

To the best of our knowledge, this is the first randomized controlled trial that evaluates the effects of laughter therapy on QOL in adult cancer patients. Our analyses detected differences in specific measures of QOL between the intervention and control groups during the initial phase using the well-established EORTC QLQ-C30.

The results showed no significant differences in global health status between the two groups at Weeks 3 or 7. However, significant between-group differences were found in two domains, although it should be noted that multiple testing increases the risk of Type I errors. First, the intervention group showed statistically significant improvements in cognitive functioning relative to baseline during the initial phase when compared with the control group. Second, participants receiving laughter therapy also had consistently greater mean benefits for pain within 3 to 7 weeks after baseline when compared with the control group. Notably, participants in the control group experienced worsening in these domains during the same period, although this may simply be due to regression to the mean. These findings suggest that laughter therapy may have physical and psychological effects in cancer patients. In addition, no negative impact of laughter therapy was detected by the QLQ-C30 scale, and the completion rates of the questionnaires were high in both groups. Laughter therapy therefore represents a potential non-pharmacological, complementary treatment for cancer patients with little or no expected harmful effects. However, these results must be considered exploratory since they were formed with no a priori hypothesis as the iOSACA study’s secondary endpoint. Furthermore, no adjustments were made for multiple statistical tests assessing a large number of domain changes.

With regard to the mechanism of cognitive function improvement, the positive emotions induced or accompanied by laughter may have enabled patients to reduce the stress response and ease tension by decreasing stress-making hormones such as cortisol, epinephrine, and growth hormone; this in turn can have a positive effect on the cognitive functioning of patients [18]. For the alleviation of pain, previous studies have reported that laughter therapy increases pain tolerance and reduces pain perception through physiological mechanisms for analgesia involving the release of endorphins [21–24, 46]. Our findings from the primary QOL analyses using results obtained during the four laughter sessions are supported by an earlier study demonstrating that even a single one-hour session of laughter therapy was able to reduce pain after a week [23].

It is interesting that participants originally randomized to laughter therapy and had experienced less appetite loss scores during the initial phase showed subsequently poorer scores in these domains at Week 11 after crossing over to the control group. The phenomenon where Group A participants showed a return to baseline QOL scores after treatment cessation suggests that the improved or stable QOL statuses may be transient, particularly in the symptom domains. This indicates that the effects of laughter therapy may be short term, although patients who continue to undergo therapy could demonstrate better preservation or slower degradation of QOL. In contrast, it is also possible that the carry-over effects are not short term. This is because no between-group differences were observed in any of the domains during the cross-over phase, where current effects and carry-over effects of laughter therapy would be observed. Notably, the slopes of Groups A and B in the score changes for emotional functioning, role functioning, and cognitive functioning between Weeks 11 and 15 were approximately parallel. A possible explanation for these findings is that the present trial design did not provide a break period after the initial phase. Also, carry-over effects may be more likely to occur in functional domains than symptom domains. We also suggest that patients may not benefit from an earlier intervention with laughter therapy soon after cancer diagnosis.

Our analysis found only two statistically significant between-group differences in the functional and symptom domains, and no significant differences in global health status. These may be explained by several reasons. First, the participants enrolled in this trial exhibited relatively good QOL status and stable disease at baseline. Such patients are likely to have ceiling effects in which improvements in QOL are less common. Second, the EORTC QLQ-C30 may not be sensitive enough to detect changes in QOL over time in patients with stable disease. For example, this questionnaire contains items related to side effects of cancer treatment (e.g., nausea and vomiting) that would have little relevance to a relatively healthy cohort of cancer survivors. As previously noted, the QLQ-C30 may not be appropriate for survivors as it was designed to capture the immediate effects of cancer treatment rather than issues related to re-integration and the long-term sequelae of treatment [47]. Third, the uneven participant characteristics between the two groups in this study should be examined in further detail. More participants in Group A had poorer baseline physical status than in Group B. This is because the study participants were not stratified with respect to PS, which may have resulted in imbalances within this small subset of participants. However, it is difficult to judge which group the imbalances favored.

Our study has several limitations that should be noted. First, as in any trial assessing patient-reported outcomes such as QOL, there may be a response shift where patients revise their internal standards or understanding of the outcomes after undergoing changes in health states. Such response shifts can affect or distort QOL outcome measurements. Second, there may be a recall bias in favor of laughter therapy. The QLQ-C30 requests patients to base their evaluations on QOL statuses in the preceding week. Because recall is influenced most strongly by a person’s current status [48], study participants may tend toward under-reporting the severity of past problems. This tendency to emphasize the current status over the past is of particular concern when evaluating outcomes immediately after an intervention. Third, there may be a social desirability bias due to the open-label nature of this study. The bias can take the form of participants tending to answer questions in a way that will be viewed favorably by others. Fourth, we were unable to determine if the effects of laughter therapy were attributable to laughter yoga or the *Rakugo* and *Manzai* performances because the study was not designed to distinguish between the effects of each type of therapy. Fifth, we did not quantitatively measure laughing during the therapy sessions. The measurement of participants’ laughter should be included in further studies for a more detailed analysis of the effects of laughter therapy. Sixth, although laughter therapy appeared to have positive effects, it should be recognized that these results may have been influenced by regression to the mean. Finally, the generalizability of the findings may be limited due to self-selection bias as many participants may have been more amenable to laughing than the overall population of cancer patients. We cannot conclude that laughter therapy would have positive effects on all cancer patients as there is scarce evidence to show these effects on individuals with a more negative disposition. Furthermore, there may be a selection bias with regard to sex distribution as most of the participants were women. The uneven sex distribution may be because men (who have a much higher employment rate than women in Japan) had more difficulty participating in the laughter therapy sessions, which were performed on weekday afternoons. This may therefore affect the generalizability of our findings to men. A previous study reported that women tend to have a greater response to laughter therapy than men [20], which may be because women have a higher tendency to laugh [49].

## Conclusions

This exploratory study provided some evidence that laughter therapy may improve specific domains of QOL and symptoms in cancer survivors. As laughter therapy has few, if any, harmful side effects, we propose that it can be implemented as a complementary therapy for cancer patients even if the beneficial effects are subtle. Additional analyses on the economic costs (including the opportunity costs of other activities) of this therapy will provide further insight into its value. However, QOL constitutes one of the secondary endpoints of this trial, and further studies are needed to verify the hypotheses generated from this exploratory study.

## Supporting information

S1 File CONSORT checklist. (DOC)

S2 File Study protocol in Japanese. (PDF)

S3 File Study protocol (English translation). (DOCX)

S4 File Relevant data underlying the findings described in manuscript. (CSV)

S5 File Laughter therapy session protocol. (PDF)
