# Supplementary material for: Effects of laughter therapy on quality of life in patients with cancer: An open-label, randomized controlled trial
Source: PLoS One. 2019 Jun 27;14(6):e0219065. doi: 10.1371/journal.pone.0219065 (PMC6597115; doi:10.1371/journal.pone.0219065)
Supplement: S2 File — (PDF) [file pone.0219065.s002.pdf]

医学研究計画書　　　　　　　　　　(Ver.28.1)

がん医療における定期的な「笑い」の提供が自己効力感や生活の質に与える効果の検証
（Initiative On Smile And CAncer, iOSACA）

１．背景

がん患者は、診断、治療、そして、がんサバイバーとしてのフォローアップの過程において、多大な身体的・精神的負担を強いられる。治療に伴う身体的有害事象と比較して、心理的や社会的な対策については置き去りになりがちであり、身体的だけでなく、精神的側面に対しても支援することにより、がんやがん治療に伴う身体的・精神的苦痛を軽減し、生活の質（Quality of Life、QOL）を維持することが重要である。QOL は、がんを受容しながら、いかに充実感や満足感を伴った生活を送ることができているかを患者が主観的に判断する評価、すなわち、がん患者目線での評価の指標であり、がん患者の生存期間と関連することが報告されている。

身体的問題や日常生活での問題に対応できているかどうかは、心理的状態にも大きな影響を及ぼす。そのような心理的な見込み感が「自己効力感」である。Lev らは、がん患者を対象とした研究において、QOL が低下すると自己効力感も低下することを明らかにし、自己効力感を高めるような支援をしていくことが QOL の維持・向上につながることを示唆している [1)]。井上らによれば、ポジティブな感情は自己効力感を高め、「笑い」はポジティブな感情を喚起する [2)] 。

笑いは、がん患者を含む病気療養者において精神的ストレスを軽減し、免疫機能を増強するとされ、Takahashi らは、笑いの免疫学的効果は、笑顔の表出の大きさではなく、楽しい気分状態や笑いの主観的経験に基づくものだと述べている [3)]。笑いは一度に限定されたものではないのが望ましいと考えられるが、これまでの笑いに関する研究は、笑い体験前後の比較に留まる。

そこで、本研究では、「笑い」の舞台を定期的に鑑賞することが、自己効力感、QOL 指標、免疫機能に与える影響を明らかにする。がん医療においては、がん患者に接する医療提供者も身体的・精神的負担を強いられていることから、がん患者のみならず、がん患者に接する医療従事者も対象とした。

２．目的と意義（研究の科学的合理性の根拠を含める）

「笑い」の舞台を定期的に鑑賞することが、がん患者やがん患者に接する医療従事者の自己効力感（主要評価項目）、QOL 指標、免疫機能に与える影響を明らかにする。

３．実施体制

大阪国際がんセンターにおいて実施する。

個人情報管理責任者（研究総括責任者）：　病院長　左近賢人

研究事務局（笑いとがん医療の実証研究会）：がん予防情報センター企画調査課　課長　宮代　勲

４．研究者等の氏名

研究総括責任者：病院長　左近賢人

研究者代表：がん予防情報センター企画調査課　課長　宮代　勲

研究協力者：

看護部　看護部長　笹田友恵、副看護部長　北坂美津子、専門看護師　高野将宏

がん予防情報センター企画調査課　課長補佐　森島敏隆

研究所腫瘍免疫学部門　部門長　井上徳光、主任研究員　赤澤　隆

臨床検査科　井戸田篤、事務局　事務局長　植田剛司

監修：福島県立医科大学　教授　大平哲也、元読売テレビプロデューサー　有川　寛

５．方法

１）デザイン：（１）ランダム化比較試験、（２）前方視的観察研究

２）登録方法：

「笑い」の舞台のスケジュールは2週間に1回の頻度で、合計8回開催し、前半4回を鑑賞する群を「A群」、後半4回を鑑賞する群を「B群」、全8回を鑑賞する群を「C群」とする。

（１）　ランダム化比較試験への同意者を対象に、属性（がん患者・医療従事者）、性、がん種で層別し、最小化法でA群かB群に無作為割付する。

（２）　ランダム化比較試験には同意が得られないが、全8回鑑賞の前方視的観察研究に同意が得られたがん患者をC群とする。

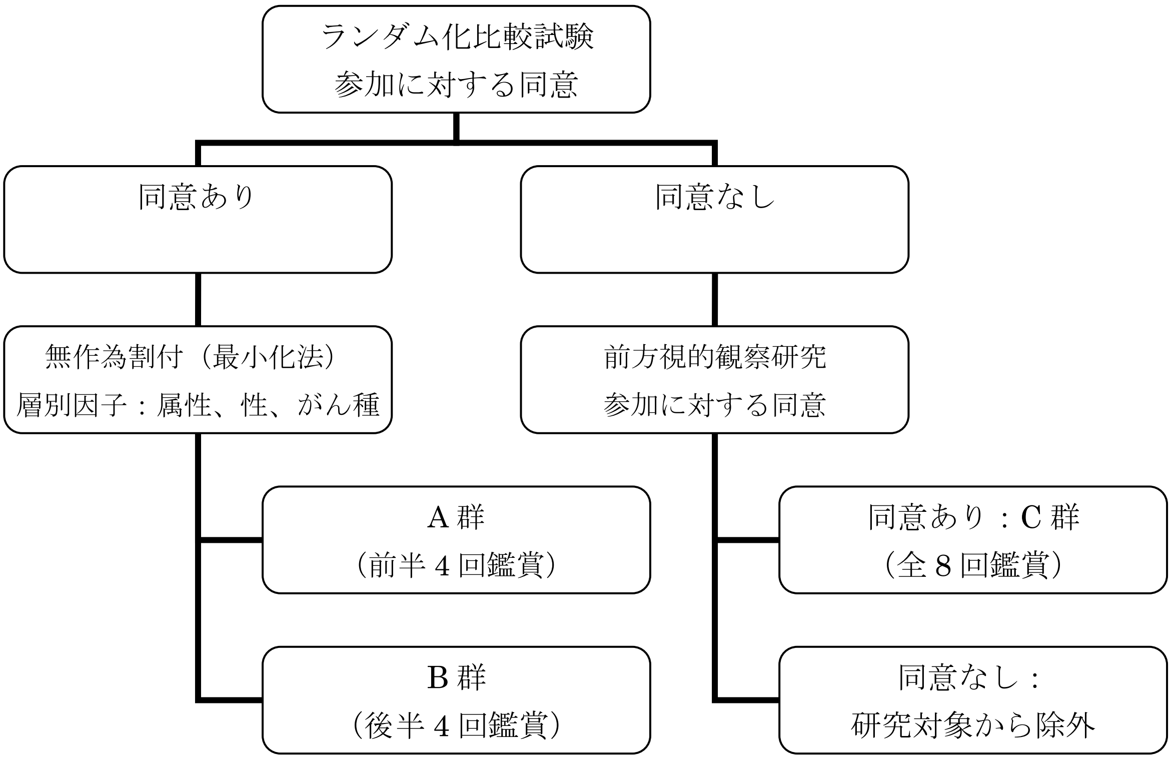

３）研究方法：

（１）　舞台鑑賞チケットと 5 回の「がん患者の健康関連 QOL 調査票（QOL 調査票）」「がん患者の病気に対する効力尺度（効力尺度用紙）」、A 群と B 群には 4 回の、C 群には 8 回の「気分尺度（TMS）」、「表情選択」をセットした冊子を作成する。舞台鑑賞チケットについては研究参加者が保持し、「笑い」舞台鑑賞時に持参する。

（２）　「笑い」舞台鑑賞日に、被験者よりチケットを回収し, 鑑賞前に血圧と脈拍を測定する。冊子から、「QOL 調査」、「自己効力尺度」、「気分尺度（TMS）前・後」、「表情選択」の用紙をバインダーにセットして研究参加者に渡し、「気分尺度（TMS）前」の記載を求める。鑑賞後に、「気分尺度（TMS）後」「表情選択」を記載し、回収箱で回収する。

（３）　採血項目は、基本的に、通常のがん診療として行われる血液検査検体を用いて測定する。

（４）　A 群、B 群、C 群とは別にコントロール群をおく（「6. 対象症例選択基準」参照)。

【スケジュール】A 群：◎●△、B 群：◎○△、C 群：◎●○△（△：検体がない場合はスキップ可）

＊ ベースライン（鑑賞回 0）を除き、鑑賞後に所見をとる。

鑑賞回		0	1		2		3		4		5		6		7		8	
週			1	2	3	4	5	6	7	8	9	10	11	12	13	14	15	16
所見	患者背景	◎																
一般	Performance Status (PS)	◎																
	血圧・脈拍（鑑賞前）		●		●		●		●		○		○		○		○	
調査	表情選択*		●		●		●		●		○		○		○		○	
	気分尺度（前・後）		●		●		●		●		○		○		○		○	
	QOL 調査票*	◎			◎				◎				◎				◎	
	効力尺度*	◎			◎				◎				◎				◎	
血液	免疫機能*	◎			△				◎				△				◎	
	コルチゾール*	◎			△				◎				△				◎	
	白血球数・リンパ球数*	◎			△				◎				△				◎	

４）目標症例数：がん患者が家族を同伴することが多いこと、会場の収容人数制限から、A 群と B 群はがん患者と医療従事者が各 30 人、C 群はがん患者 30 人とする（計 150 人）。各測定値を比較検討する（がん患者と医療従事者別解析を行う）。コントロール群は 5 名とする。

竹嶋らによれば、外来通院血液腫瘍患者 70 名の自己効力感尺度の平均値は 26.8±標準誤差 5.0 であり[4)]、これをがん患者のベースラインの自己効力感尺度と仮定すると、本研究の非介入 30 例の平均値は 26.8±標準誤差 7.64、95%信頼区間が 11.8-41.8 と推定される。「笑い」の定期的介入による自己効力感尺度の増加に関する先行研究はないため、効果サイズの推定は困難であるが、坂野によれば、一般成人の自己効力感尺度は 50.1、三澤らによれば、看護師の自己効力感尺度は 33.8 と報告されている[5)6)]。

５）付随研究：付随研究として、（１）血液検査検体を用いたより詳細な免疫機能の測定、（２）「笑い」の舞台を鑑賞する本研究参加者以外のアンケート協力者に対するアンケート調査を検討する。

６．対象症例選択基準（被験者の選定方針）

（１） 対象者

がん患者：がんの部位を問わない。化学療法や放射線療法を受けている症例など。

医療従事者：がん患者に日常的に接する看護師など。

なお、コントロール群として同意が得られた、事務職員などの非がん患者かつ（狭義の）非医療従事者に対して、測定系安定目的の鑑賞回 0 以前の免疫機能、比較コントロールとしての鑑賞回 0,4,8 のコルチゾールを除く血液所見をとる。

（２） 選択基準

【がん患者】

① 組織学的に悪性腫瘍と診断が得られている

② 年齢 40 歳以上 65 歳未満である

③ 同意取得時に Performance Status (PS) が ECOG 基準 0-2 である

④ １年以上の生存が可能と担当医が判断している

【医療従事者】

① 病棟もしくは外来でがん患者に日常的に接している常勤職員である

② 年齢 20 歳以上 65 歳未満である

（３）除外基準

① アンケート用紙の内容が理解できない

② 自身で読み書きができない

③ 研究への継続的参加が困難になると予想される（例：期間中に手術を受ける等）

７．研究期間：倫理審査委員会通過後から 2018 年 3 月 31 日まで

８．検査及び評価項目

（１） 自己効力感尺度：平井らが作成したもので、質問項目は 18 項目からなり「全く自信がない：0」から「完全に自信がある：100」の 11 件法の Numerical Rating Scale で評価する [7]。下位因子「情動統制に対する効力感」、「症状コントロールに対する効力感」、「日常生活動作に対する効力感」から構成され、因子間の相関が認められている尺度全体の得点の平均点（100 点満点）を自己効力得点として判断可能である。得点が高いほど自己効力感が高いことを示す。

（２） 健康関連 QOL 調査票（EORTC QLQ-C30 日本語版）：Aaronson らにより開発されたもので [8]、日本語版 QLQ-C30 は総括的な QOL と 5 つの活動性尺度（身体的活動性・役割活動性・社会的活動性・精神的活動性・認識する活動性）と 8 つの症状尺度（疲れ・悪心・嘔吐・痛み・

息切れ・不眠・食欲不振・便秘・下痢)、経済状態の 15 項目からなり、30 の質問により点数化し、EORTC のスコアリングマニュアルに従いスコア化して QOL を評価する。活動性尺度と総括的 QOL スコアについては高得点ほど良い QOL 状態であり、身体症状尺度/項目と経済状況では高得点ほど悪い状態を示す。

（３）　一時的気分尺度 (Temporary Mood Scale) : 徳田の報告[9)10)]を参考に作成されたもので、「緊張」、「抑鬱」、「怒り」、「混乱」、「疲労」、「活気」の 6 因子からなる 18 項目、今現在の気分状態について「1 : 全く当てはまらない、2 : あまり当てはまらない、3 : どちらでもない、4 : やや当てはまる、5 : かなり当てはまる」のいずれかを選択してもらう 5 件法である。

（４）　表情選択法 : Face Scale 法を簡略化したもので、5 段階の表情図形の中からどのくらい笑ったかの笑いのレベルを選択する。

（５）　採血項目 : 白血球数とリンパ球数については、通常のがん診療として行われた検査結果を用いる。免疫機能とコルチゾールについては、通常のがん診療として行われる血液検査の検体を用いて測定する（採血量が約 5ml 増えると推定される)。免疫機能として、活性化免疫細胞（NK 細胞、T 細胞等）や抑制性免疫細胞（制御性 T 細胞や抑制性骨髄系細胞等）の数的な変化を、細胞膜表面抗原や細胞内抗原を用いて FACS で測定する。がんの免疫療法のターゲットとなる免疫チェックポイント分子（PD-1、PD-L1、CTLA-4）を発現する細胞数についても測定する。また、免疫細胞を免疫刺激物質 (PHA,や LPS 等) で刺激し、サイトカイン産生能 (IFNγ、IL10、IL12 等）を評価する。

９．期待される利益と起こり得る危険（予期される有害反応など）及び必然的に伴う不快な状態

期待される利益としては、がん医療において、自己効力感が高まることで、QOL が維持・向上する可能性があることで、少なくとも、「笑い」の舞台の定期的鑑賞が、がん患者やがん患者に接する医療従事者の自己効力感、QOL 指標、免疫機能に与える影響を明らかにすることで、今後の定期的な笑いの場の提供の是非が示唆される。

起こり得る危険（予期される有害反応など）及び必然的に伴う不快な状態としては、治療中および通院と異なる時間帯および日程に「笑い」の舞台鑑賞が行われることと継続的な参加を要することで、時間的制約と拘束感を強いる可能性がある。

１０．補償の有無

本臨床試験に参加することで生じた健康被害については、通常の診療と同様に病状に応じた適切な治療を保険診療として提供する。その際、医療費の自己負担分については患者の負担とする。見舞金や各種手当てなどの経済的な補償は行わない。

１１．緊急・救命的研究 : 該当しない

１２．　インフォームドコンセントのための手続き : 本研究の説明・同意文書を用いる。

１３．インフォームドコンセントを受けるための説明事項：別添

１４．相談等への対応

研究に関する問い合わせ、苦情等の窓口

大阪府立成人病センター　研究所病院共同研究連携室

室長　片山　和宏

住所：〒５３７－８５１１　大阪市東成区中道１－３－３

Fax：０６－６９７７－６１５１

e-mail：kbyori01@mc.pref.osaka.jp

１５．研究実施後における医療の継続提供の有無：該当しない

１６．重篤な有害事象発現時の対応：該当しない

１７．総長への報告

研究の進捗状況及び研究実施に伴う有害事象の発生状況を原則年１回、文書等で報告する。また、研究の倫理的妥当性若しくは科学的合理性を損なう事実もしくは情報又は損なうおそれのある情報であって研究の継続に影響を与えると考えられるものを得た場合（次に該当する場合を除く。）には、遅滞なく報告する。研究の実施の適正性もしくは研究結果の信頼を損なう事実もしくは情報または損なうおそれのある情報を得た場合には、速やかに報告する。研究を終了（中止の場合を含む）したときには報告する。

１８．個人情報保護の方法

収集したデータは本研究のみに使用し、個人が特定できないような形で提示し、個人の秘密は厳守される。研究データは研究のみに使用し、匿名性を保持するために個人名がわからないように配慮し、管理する。収集したデータに関する記録物は研究完了の後（論文等の発表から10年以上経過後）、研究総括責任者の確認の下で処分する。

１９．情報公開の方法：研究開始までにUMIN-CTRに登録を予定している。

２０．試料・情報の保管及び廃棄

本研究で使用される試料は、検査部門内で保管され、検査項目のデータ収集が終了した時点で、医療廃棄物として廃棄する。

２１．医学研究に係る資金源：大阪国際がんセンターが負担する。

２２．利益相反：なし

２３．経済的負担又は謝礼：該当しない。この研究に関する費用のうち、鑑賞および通常のがん診療として行われる項目以外の検査の費用負担を参加者には求めない（交通費は参加者自己負担）。

２４．被験者の健康、子孫に受け継がれる遺伝的特徴に関する重要な知見が得られる可能性：該当しない

２５．業務の委託：該当しない

２６．　モニタリング・監査の体制と実施方法：　研究が適正に行われることを確保するため、研究がどの程度進捗しているか並びに研究計画書に従って行われているか（モニタリング）、研究結果の信頼性を確保するため、研究が研究計画書に従って行われたか（監査）、別紙「モニタリングの実施に関する手順書」に従い、研究総括責任者が指定した者により調査が行われる。

【文献】

1. Lev EL, Owen SV. A measure of self-care self-efficacy. Res Nurs Health. 1996;19:421-9.
2. 井上宏：笑いは心の治癒力．海竜社，東京，1997
3. Takahashi K et al. The elevation of natural killer cell activity induced by laughter in a crossover designed study. Int J Mol Med. 2001;8:645-50.
4. 竹嶋順平，楠葉洋子，浦田秀子：外来に通院している血液疾患患者の QOL とその関連要因．保健学研究．2009;22,9-15.
5. 坂野雄二：一般性セルフ・エフィカシー尺度の妥当性の検討．早稲田大学人間科学研究．1989;2, 91-98.
6. 三澤寿美，遠藤恵子：成人女性の自己効力感に関する研究．山形保健医療研究．2002;5, 37 -42.
7. Hirai K., et al. A structural model of the relationships among self-efficacy, psychological adjustment, and physical condition in Japanese advanced cancer patients. Psychooncology. 2002;11:221-9.
8. Aaronson NK, et al. The European Organization for Research and Treatment of Cancer QLQ-C30: a quality-of-life instrument for use in international clinical trials in oncology. J Natl Cancer Inst. 1993;85:365-76.
9. 徳田完二．筋弛緩法による気分変化．立命館人間科学研究．2007;13:1-7.
10. 徳田完二．一時的気分尺度（TMS）の妥当性．立命館人間科学研究．2011;22:1-6.

医学研究　参加同意書

大阪国際がんセンター総長　　様

医学研究名：
<u>がん医療における定期的な「笑い」の提供が自己効力感や生活の質に与える効果の検証</u>

説明内容：

☐　医学研究の目的と方法
☐　予想される効果と起こりうる危険
☐　医学研究への参加は自由意志によること
☐　研究に同意しない場合でも不利益を受けないこと
☐　同意した後でも随時これを撤回できること
☐　個人情報が充分に保護されていること
☐　医学研究の研究計画書等を閲覧することができること
☐　問い合わせあるいは苦情窓口があること
☐　その他

上記の医学研究について、担当医師から説明を受け、理解しましたので、その参加することに同意します。

研究参加者署名：＿＿＿＿＿＿＿＿＿＿＿＿＿＿＿＿＿＿＿＿＿＿＿＿

署名年月日：　　　平成　　年　　月　　日

私は、今回の医学研究について上記項目を説明し、同意が得られたことを認めます。

担当医師署名：＿＿＿＿＿＿＿＿＿＿＿＿＿＿＿＿＿＿＿＿＿＿＿

署名年月日：　　　平成　　年　　月　　日

医 学 研 究 同 意 撤 回 書

大阪国際がんセンター総長　　様

医学研究名：
がん医療における定期的な「笑い」の提供が自己効力感や生活の質に与える効果の検証

私は、上記の医学研究に協力することに同意しましたが、
同意を撤回しますので通知します。

研究参加者署名：＿＿＿＿＿＿＿＿＿＿＿＿＿＿＿＿＿＿（署名）

署名年月日：　　　平成　　年　　月　　日

代諾者氏名：＿＿＿＿＿＿＿＿＿＿＿＿＿＿＿＿（署名）

代諾者と本人との関係：＿＿＿＿＿＿＿＿＿＿＿＿

署名年月日：　　　平成　　年　　月　　日
