# Supplementary material for: Effects of laughter therapy on quality of life in patients with cancer: An open-label, randomized controlled trial
Source: PLoS One. 2019 Jun 27;14(6):e0219065. doi: 10.1371/journal.pone.0219065 (PMC6597115; doi:10.1371/journal.pone.0219065)
Supplement: S3 File — (DOCX) [file pone.0219065.s003.docx]

Initiative On Smile And CAncer (iOSACA)

Lead investigator:

Isao Miyashiro, MD PhD

Cancer Control Center, Osaka International Cancer Institute

3-1-69 Otemae, Chuo-ku, Osaka 541-8567, Japan

Tel +81-6-6945-1181

Email miyashir@biken.osaka-u.ac.jp

Registered date:

April 3, 2017

**Study protocol**

**Basic information**

Official scientific title of the study: Initiative On Smile And CAncer

Title of the study (Brief title): iOSACA

Region: Japan

**Condition**

Subjects: Cancer patients, healthcare professionals, or hospital administrative staff

Classification by specialty: Hematology and clinical oncology, Gastrointestinal surgery, Thoracic surgery, Breast surgery, Obstetrics and gynecology, Otorhinolaryngology, Urology, Nursing

Classification by malignancy: Malignancy

Genomic information: No

**Objectives**

Narrative objectives: Impact of periodic laughter on the self-efficacy of cancer patients or healthcare professionals.

Basic objectives: Safety, Efficacy

**Assessment**

Primary outcome: self-efficacy (measured by “Self-efficacy scale for Advanced Cancer”)

Secondary outcome: quality of life (measured by “EORTC QLQ-C30”)

Secondary outcome: mood (measured by “Temporary Mood Scale” and “Face Scale”)

Secondary outcome: immune profile (measured by immune cell subsets and cytokine producibility)

**Base**

Study type: Interventional

**Study design**

Basic design: Cross-over

Randomization: Randomized

Randomization unit: Individual

Blinding: Open, but assessor(s) are blinded

Control: No treatment

**Intervention**

No. of arms: 3

Purpose of intervention: Treatment

Type of intervention: Behavioral, custom

Interventions/Control_1: Periodic laughter therapy sessions

Interventions/Control_2: No periodic laughter therapy sessions

Interventions/Control_3: Periodic laughter therapy sessions (non-randomized, prospective observational study)

**Eligibility**

Key inclusion criteria: Cancer patients

1. Histologically proven cancer

2. Aged 40–64 years

3. Eastern Cooperative Oncology Group performance status 0–2

4. Expected survival time ≥1 year

Key inclusion criteria: Healthcare professionals

1. Permanent staff, contact with cancer patients on a daily basis

2. Aged 20–64 years

Key exclusion criteria

1. Unable to comprehend questionnaires

2. Illiteracy

3. Lack of continuity

Target sample size: 60 for the randomized double-arm trial of cancer patients, 30 for the single-arm trial of cancer patients, 60 for the randomized double-arm trial of healthcare professionals, and 5 for the single-arm trial of hospital administrative staff

**Funding Source**

Organization: Osaka International Cancer Institute

Category of Funding Organization: Self-funding

**Other administrative information**

Date of disclosure of the study information: April 3, 2017

Date of protocol fixation: March 31, 2017

**UMIN-CTR Clinical Trial Registration Information (in English)**

URL: https://upload.umin.ac.jp/cgi-open-bin/ctr_e/ctr_view.cgi?recptno=R000030790
