# Supplementary material for: Effects of laughter therapy on quality of life in patients with cancer: An open-label, randomized controlled trial
Source: PLoS One. 2019 Jun 27;14(6):e0219065. doi: 10.1371/journal.pone.0219065 (PMC6597115; doi:10.1371/journal.pone.0219065)
Supplement: S5 File — (PDF) [file pone.0219065.s005.pdf]

Laughter Therapy Session Protocol

Held at the Hall on the 1st floor, Osaka International Cancer Institute, Osaka, Japan

Week 1 (Thursday, May 18, 2017)

2:00 pm: Greeting from the hospital president, director, or investigator

2:05 pm: Laughter yoga

2:15 pm: *Rakugo* Part 1

2:25 pm: *Rakugo* Part 2

2:40 pm: *Rakugo* Part 3

3:00 pm (end of the session)

Week 3 (Thursday, June 1, 2017)

2:00 pm: Greeting from the hospital president, director, or investigator

2:05 pm: Laughter yoga

2:15 pm: *Manzai* Part 1

2:30 pm: *Manzai* Part 2

2:45 pm: *Manzai* Part 3

3:00 pm (end of the session)

Week 5 (Thursday, June 15, 2017)

2:00 pm: Greeting from the hospital president, director, or investigator

2:05 pm: Laughter yoga

2:15 pm: *Manzai* Part 1

2:25 pm: *Manzai* Part 2

2:40 pm: *Manzai* Part 3

3:00 pm (end of the session)

Week 7 (Thursday, June 29, 2017)

2:00 pm: Greeting from the hospital president, director, or investigator

2:05 pm: Laughter yoga

2:15 pm: *Rakugo* Part 1

2:25 pm: *Rakugo* Part 2

2:35 pm: *Rakugo* Part 3

2:45 pm: *Rakugo* Part 4

3:00 pm (end of the session)

Week 9 (Thursday, July 13, 2017)

2:00 pm: Greeting from the hospital president, director, or investigator

2:05 pm: Laughter yoga

2:15 pm: *Manzai* Part 1

2:25 pm: *Manzai* Part 2

2:40 pm: *Manzai* Part 3

3:00 pm (end of the session)

Week 11 (Thursday, July 27, 2017)

2:00 pm: Greeting from the hospital president, director, or investigator

2:05 pm: Laughter yoga

2:15 pm: *Manzai* Part 1

2:25 pm: *Manzai* Part 2

2:40 pm: *Manzai* Part 3

3:00 pm (end of the session)

Week 13 (Thursday, August 10, 2017)

2:00 pm: Greeting from the hospital president, director, or investigator

2:05 pm: Laughter yoga

2:15 pm: *Rakugo* Part 1

2:25 pm: *Rakugo* Part 2

2:35 pm: *Rakugo* Part 3

2:45 pm: *Rakugo* Part 4

3:00 pm (end of the session)

Week 15 (Thursday, August 24, 2017)

2:00 pm: Greeting from the hospital president, director, or investigator

2:05 pm: Laughter yoga

2:15 pm: *Rakugo* Part 1

2:35 pm: *Rakugo* Part 2

3:00 pm (end of the session)

The laughter yoga sessions were led by a professional laughter yoga therapist. Investigators were present throughout the therapy sessions. The *Rakugo* and *Manzai* comedy sessions were conducted by a variety of professional performers dispatched by a local entertainment agency; the content of the comedy performances was decided by each performer.
